# Identification of di-substituted ureas that prevent growth of trypanosomes through inhibition of translation initiation

**DOI:** 10.1038/s41598-018-23259-9

**Published:** 2018-03-20

**Authors:** Fabricio Castro Machado, Caio Haddad Franco, Jose Vitorino dos Santos Neto, Karina Luiza Dias-Teixeira, Carolina Borsoi Moraes, Ulisses Gazos Lopes, Bertal Huseyin Aktas, Sergio Schenkman

**Affiliations:** 1 Departamento de Microbiologia, Imunologia e Parasitologia, Escola Paulista de Medicina, Universidade Federal de São Paulo, 04039-032 São Paulo, SP Brazil; 2 Laboratório de Parasitologia Molecular, Instituto de Biofísica Carlos Chagas Filho, Universidade Federal do Rio de Janeiro, Rio de Janeiro, RJ Brazil; 3 Instituto Butantan, São Paulo, SP Brazil; 4 Departamento de Microbiologia, Instituto de Ciências Biomédicas, Universidade de São Paulo, São Paulo, SP Brazil; 5 Hematology Laboratory for Translational Research, Department of Medicine, Brigham and Women’s Hospital and Harvard Medical School, 75 Francis Street, Boston, MA 02115 United States

## Abstract

Some 1,3-diarylureas and 1-((1,4-*trans*)−4-aryloxycyclohexyl)−3-arylureas (cHAUs) activate heme-regulated kinase causing protein synthesis inhibition via phosphorylation of the eukaryotic translation initiation factor 2 (eIF2) in mammalian cancer cells. To evaluate if these agents have potential to inhibit trypanosome multiplication by also affecting the phosphorylation of eIF2 alpha subunit (eIF2α), we tested 25 analogs of 1,3-diarylureas and cHAUs against *Trypanosoma cruzi*, the agent of Chagas disease. One of them (I-17) presented selectivity close to 10-fold against the insect replicative forms and also inhibited the multiplication of *T. cruzi* inside mammalian cells with an EC_50_ of 1–3 µM and a selectivity of 17-fold. I-17 also prevented replication of African trypanosomes (*Trypanosoma brucei* bloodstream and procyclic forms) at similar doses. It caused changes in the *T. cruzi* morphology, arrested parasite cell cycle in G1 phase, and promoted phosphorylation of eIF2α with a robust decrease in ribosome association with mRNA. The activity against *T. brucei* also implicates eIF2α phosphorylation, as replacement of WT-eIF2α with a non-phosphorylatable eIF2α, or knocking down eIF2 protein kinase-3 by RNAi increased resistance to I-17. Therefore, we demonstrate that eIF2α phosphorylation can be engaged to develop trypanosome-static agents in general, and particularly by interfering with activity of eIF2 kinases.

## Introduction

Parasitic diseases caused by protozoa such as Chagas disease and African Trypanosomiasis still lack effective, safe, and highly accessible chemotherapy^1–4^. The available drugs are toxic, the treatments show a high degree of abandon due to aggressive side effects^5^ and resistance has been detected^6^. Currently, very few compounds are on clinical trials for Chagas disease and African Trypanosomiasis caused respectively by *Trypanosoma cruzi* and *Trypanosoma brucei*^7,8^.

Different approaches have been devised to identify new compounds to treat these diseases. Previous works have explored unique trypanosome biological processes to find new drug targets. Examples are studies targeting cysteine proteases, farnesyl pyrophosphatase synthase, glyceraldehyde 3-phosphate-dehydrogenase, 14α-demethylase and trans-sialidases^9–11^. The enzyme 14α-demethylase has gathered attention due to its essential role in biosynthesis of steroids. This enzyme is inhibited by azolic compounds originally developed as antifungal agents, which were subsequently repurposed for treatment of Chagas disease^12,13^. Although, the treatment with a 14α-demethylase inhibitor, called posaconazole, has shown promising results, this treatment did not eliminate the parasite in most cases, probably due to differences in parasite strains^14,15^ and prompted combinatory therapies^16^.

A second approach has been to perform high-throughput screenings to identify new active compounds. For example, natural product libraries were used to identify new lead molecules against *T. cruzi*, *T. brucei* and *Leishmania*^17^. It is also noteworthy that a set of 1.8 million compounds from GlaxoSmithKline tested against these parasites revealed possibly new therapeutic molecules, including kinase inhibitors^18^.

Since drug exposure is an environmental challenge for parasites and considering that, for most cases, differences in strains susceptibility are also a problem^15^, global molecular responses to different stresses, such as the drug treatment, could be explored as a tool to improve the chemotherapy of parasitic diseases. A similar approach has been proposed for pathogenic fungi, such as *Candida albicans*^19^, where a genome-wide transcriptional factor has a dual role during stress fine-tuning responses to drug exposure and resistance. The phosphorylation of eIF2α has been recognized as the “integrated stress response hub” for various organisms, arresting general translation and promoting the accumulation of proteins and enzymes that cope with different types of damaging situations^20^. However, when the phosphorylation is sustained for long periods, apoptosis and cell death are induced^21^.

Urea derivatives, particularly those substituted by cyclic rings, provide significant opportunities for drug development and some of them have been previously shown to trigger eIF2α phosphorylation in mammalian cells^22–24^, a key event in the stress response and cell survival, especially for cancer cells chemotherapy^25^. Some urea derivatives were found to activate the heme-regulated kinase, one of the four mammalian kinases that phosphorylate eIF2α, arresting cancer cell proliferation^26^. In addition, other substituted ureas could inhibit several enzymes^27^, prevent cellular growth^28^ and interfere with activation of intracellular complexes^29^.

Here, to exam the possible use of 1,3-di-substituted ureas in Trypanosomatids and to check whether they would also interfere with stress response and phosphorylation of eIF2α, we initially evaluated the activity of 25 urea derivatives against *T. cruzi* epimastigotes, which are the free proliferating form found in the midgut of the insect vector. Three compounds were found effective in preventing parasite growth and one of them, named I-17, showed a better selectivity index, controlling the intracellular replicative form at 17-fold lower concentrations than the detected toxicity to human host cells. Therefore, we further evaluate the biological effects of I-17 treatment and used *T. brucei*, which has more available genetic tools, to investigate how eIF2α phosphorylation and which eIF2α kinases were involved in growth inhibition by I-17.

## Results

### *T. cruzi* epimastigotes replication was inhibited by 1,3-diarylureas and cHAUs

*T. cruzi* epimastigotes were incubated in culture medium for three days with 25 different compounds at 10 µM. Seventeen inhibited parasite multiplication, while other 8 (3b, 3n, 3d, 3j, 3k, 3 l, 3t and NCPdCPU) were unable to produce any visible effect at 10 µM (data not shown). Therefore, we titrated the concentrations that caused 50% (EC_50_) and 90% (EC_90_) viability inhibition for the 17 more active compounds using an Alamar Blue assay, which is based on the capacity of viable cells to reduce resazurin to resofurin. Nine compounds (3p, 6a, I-17, 3e, 6b, I-18, 3 g, 3r and 3q) were most active against epimastigotes with EC_50_ concentrations ranging from 1.3 to 3.7 ± 2.5 µM (Table 1). The other eight presented EC_50_ values higher than 5 µM. We also tested benznidazole (BZN), a reference compound for Chagas disease treatment, using the same Y strain and protocol to obtain an EC_50_ of 211 ± 3 µM. This is remarkable since even the less active of the 17 1,3-di-substituted ureas presented at least 25-fold higher activity compared to BZN using a resistant strain, demonstrating the susceptibility of *T. cruzi* to these compounds. To further select useful compounds, we determined the selectivity index by measuring viability of monkey kidney cells (LLC-MK_2_) for the most active compounds, also using Alamar Blue. As shown in Table I, the compounds were usually toxic for mammalian cells, except 3p, 6a and I-17, which were at least 5-fold more effective in damaging parasites than host cells.

**Table 1:** Effective concentration (EC) in µM of 25 di-substituted urea compounds against *T. cruzi* epimastigotes and LLC-MK_2_ cells.

Compound	*T. cruzi* epimastigotes^a^		LLC-MK_2_^b^ EC_50_ (μM)	Selectivity Index^c^ (SI)
	EC_50_ (μM)	EC_90_ (μM)		
3p	1.3^d^	4.4^d^	7.7 ± 3.7	5.9
6a	1.7 ± 0.1	7.5 ± 0.5	13.7 ± 8.7	8.0
I-17	3.4 ± 1.3	5.7 ± 1.5	32.4 ± 14.7	9.5
3e	2.3^d^	2.8^d^	6.6 ± 3.6	1.4
6b	2.5 ± 0.4	4.6 ± 0.1	2.5 ± 0.4	1.0
I-18	3 ± 0.7	6.6 ± 1.8	12.3 ± 8.8	4.1
3 g	3.3 ± 0.1	8.4 ± 2.4	7 ± 4.7	2.1
3r	3.5 ± 0.1	6 ± 0.9	8.6 ± 1.2	2.4
3q	3.7 ± 2.5	8.7 ± 1.0	1.2 ± 1.3	0.7
3 f	5.4 ± 0.2	16.8 ± 5.5	N.T.^f^	N.T.
3o	5.5 ± 0.4	7.3 ± 0.8	N.T.	N.T.
3 m	5.9 ± 0.3	7.7 ± 0.1	N.T.	N.T.
3i	6.3 ± 0.5	12.1 ± 1.7	N.T.	N.T.
I-m6	6.5 ± 0.2	10.4 ± 0.8	N.T.	N.T.
3a	7.7 ± 0.5	9.9 ± 0.3	N.T.	N.T.
3c	7.7 ± 0.4	9.7 ± 0.4	N.T.	N.T.
3 h	8.2 ± 0.1	13.5 ± 4.5	N.T.	N.T.
3b	>10^g^	>10	N.T.	N.T.
3n	>10	>10	N.T.	N.T.
3d	>10	>10	N.T.	N.T.
3j	>10	>10	N.T.	N.T.
3k	>10	>10	N.T.	N.T.
3 l	>10	>10	N.T.	N.T.
3t	>10	>10	N.T.	N.T.
NCPdCPU	>10	>10	N.T.	N.T.
BZN^e^	211 ± 3.0	960 ± 25.0	N.T.	N.T.

^a^Epimastigotes of *T. cruzi* were incubated for 3 days in the presence of different concentrations of each compound in triplicate experiments, each value  determined in duplicate measurements. The concentration that inhibited 50% (EC_50_) or 90% (EC_90_) of multiplication was determined by using the Alamar Blue assay as described in Methods.

^b^LLC-MK_2_ were plated and after 24 h, incubated with the different concentrations of the indicated compounds. After two more days, cell viability was measured with Alamar Blue assay as described in Methods and the results used to determine the (EC_50_).

^c^TI = Ratio between *T. cruzi* EC_50_ and LLC-MK_2_ EC_50_.

^d^These values correspond to a single experiment.

^e^BZN = Benznidazole.

^f^N.T. = not tested.

^g^>10 = without apparent effect at 10 µM.

### I-17 inhibited multiplication of different stages of *T. brucei* and *T. cruzi* parasites

**Figure 1:**
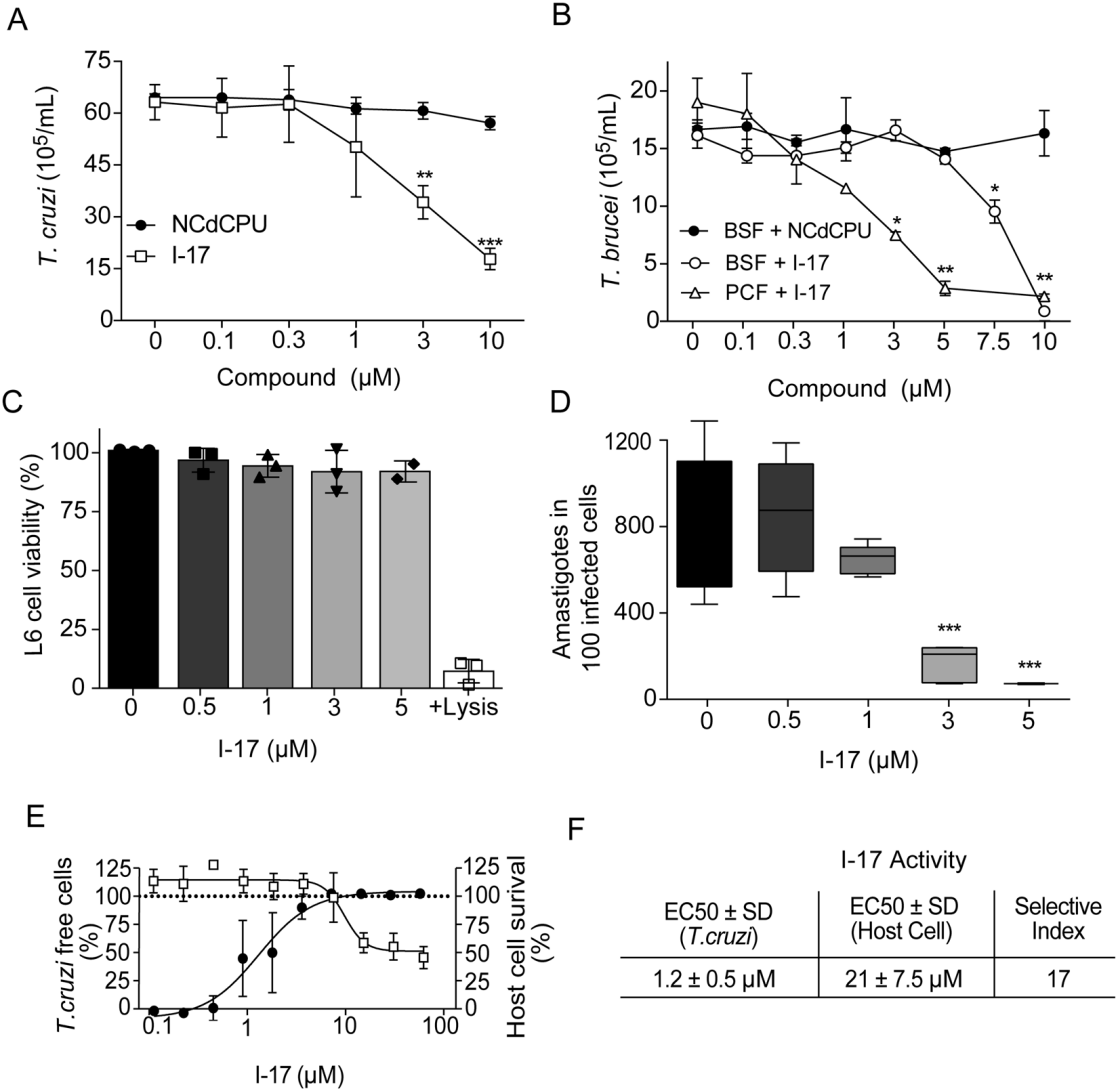
Replication of *T. cruzi* and *T. brucei* forms are inhibited by I-17. (**A**) *T. cruzi* epimastigotes were inoculated at 2 × 10^6^ parasites per mL and incubated at 28 °C in LIT medium containing the indicated concentrations of NCPdCPU (an inactive 1,3-diarylurea, circles) or I-17 (squares). The numbers are means ± standard deviation (n = 3) of parasite counted after 48 hours of incubation. (**B**) *T. brucei* BSF (circles) and PCF (triangles) were inoculated at 2 × 10^5^ parasites per mL in their respective culture media in the presence of the indicated concentrations of I-17, or NCPdCPU (closed symbols). The numbers are means ± standard deviation (n = 3) of parasites counted after 24 hours at 37 °C and 48 hours at 28 °C, respectively for BSF and PCF. (**C**) Relative viability of L6 myoblasts measured by activity of lactate dehydrogenase released after 72 hours in the presence of the indicated concentrations of I-17. (**D**) TCT was used to infect L6 cells. After infection the cells were treated for 72 hours with the indicated concentrations of I-17 and the number of intracellular amastigotes was determined by optical microscopy as described in Methods. The bars represent means ± standard deviation of three independent experiments. In all panels, *indicate p < 0.05, **p < 0.01 and ***p < 0.001 calculated using the Student’s T-test comparing to non-treated cells. (**E**) Serial dilutions of I-17 was used to treat U-2 OS cells infected with *T. cruzi* TCT. Compound activity is indicated as percentage of parasite free cells (black circles) and the host cell survival (empty squares), both calculated as described in Methods. (**F**) Calculation of EC_50_ values for *T. cruzi* and host cells expressed in µM are also explained in Methods. The values in (**E**) and (**F**) represent means ± standard deviation of three independent experiments.

As I-17 was the best among the 25 tested compounds, with a selectivity index of 9.5-fold over LLC-MK_2_ cells, we decided to further characterize its effects on different proliferative stages of *T. cruzi* and *T. brucei*. *T. cruzi* epimastigotes multiplication was diminished by 50% at ~3 µM I-17 (Fig. 1A), a value close to 3.4 µM, the EC_50_ concentration obtained by the Alamar Blue assay (Table I). In contrast, minimal inhibition was observed for all tested concentrations of NCPdCPU, one inactive 1,3-diarylureas (EC_50_>10 µM). We also observed that up to 10 µM, I-17 could be washed out and the parasites would re-start to multiply (data not shown), indicating that it has a trypanostatic effect on epimastigotes. We also tested the effect of I-17 in cultures of *T. brucei* bloodstream form (BSF) that corresponds to the proliferative stage in mammalian host blood, and cultures that contained the procyclic form (PCF), the stage found in the gut of the insect vector. The multiplication of both forms was inhibited by I-17 but not by NCPdCPU (Fig. 1B). The inhibitory effect was more pronounced in PCF, starting to be detected at concentrations as low as 3 µM.

We observed that L6 rat myoblasts were also marginally affected after 72 hours incubation with up to 5 µM I-17, using a lactate dehydrogenase activity assay as a biomarker for cytotoxicity and cytolysis (Fig. 1C). Furthermore, we tested whether I-17 could also inhibit the proliferation of *T. cruzi* amastigotes in infected L6 cells. As shown in Fig. 1D, amastigote replication was strongly impaired when cells were treated with 3 µM I-17 for 72 h. These results indicated that I-17 prevented replication of different stages of the two *Trypanosoma* species.

To further validate the selectivity in *T. cruzi* intracellular forms, we infected U-2 OS cells plated on 384 well-microplates. The cells were treated with different concentrations of I-17 for 96 hours and the number of infected, non-infected cells and amastigotes was determined through high content analysis, which enables automated quantification of cells and parasites based on DNA staining (Fig. 1E). We found that I-17 caused a decrease in the numbers of *T. cruzi* amastigotes inside infected cells, with an EC_50_ of 1.2 ± 0.5 µM (Fig. 1F). In the same cultures, the host cell cytotoxicity was evaluated by counting the total number of cells in treated wells relative to the number of cells in non-treated wells, showing that I-17 was mildly toxic to host cells (EC_50_ = 21 ± 7.5 µM).

### I-17 changes trypanosomatids morphology and cell cycle

**Figure 2:**
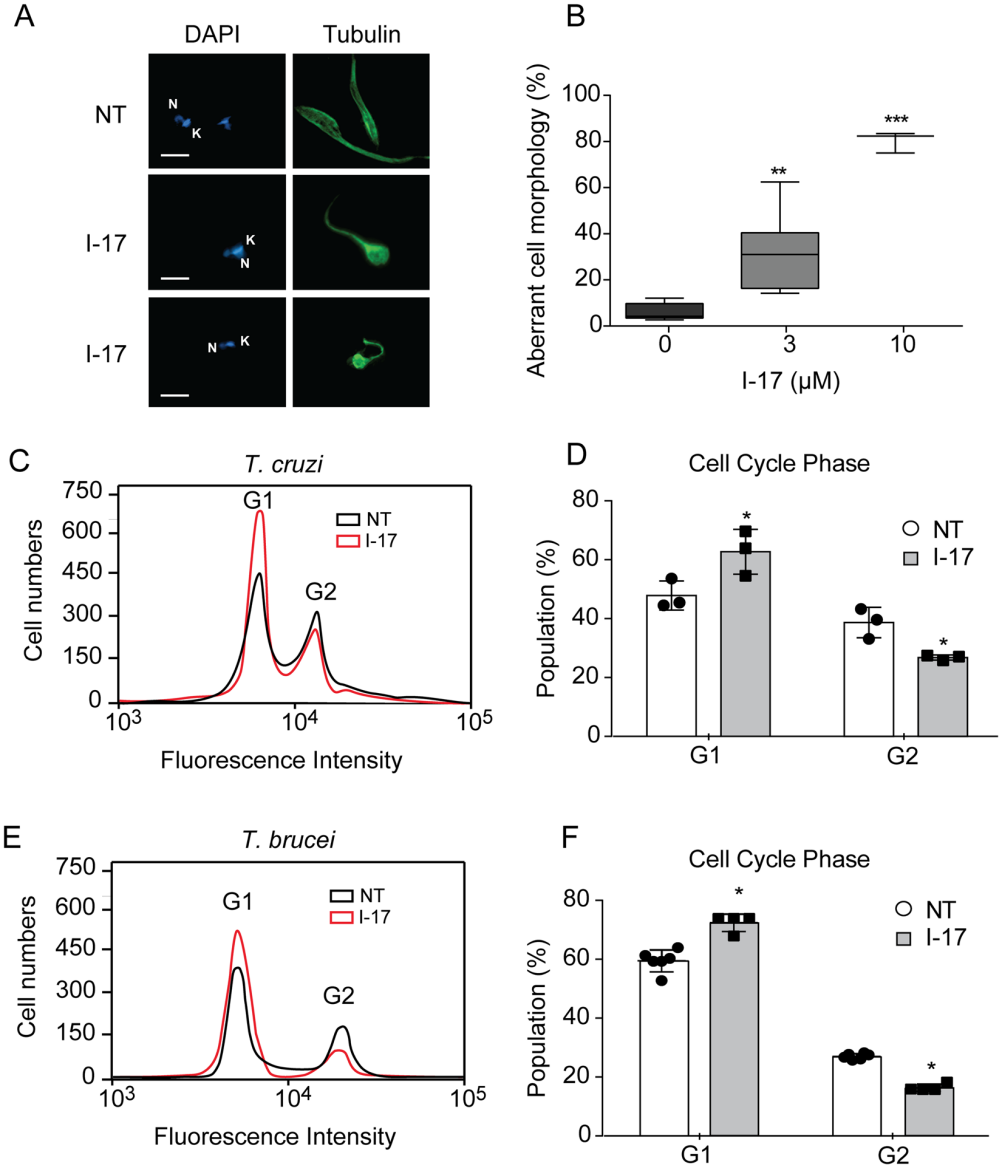
I-17 treatment causes aberrant morphology and G1 cell cycle arrest. (**A**) Epimastigotes non-treated (NT) or treated with 3 µM of I-17 for 4 hours were immunostained for β-tubulin (green) and labeled with DAPI to show the nucleus (N) and kinetoplast (K) (blue). Bars = 5 µM. (**B**) Percentage of epimastigotes with aberrant morphology after 4 hours treatment with the indicated concentrations of I-17. Boxes are representation of median ± max and min values. **p < 0.01 and ***p < 0.001 were calculated using Mann-Whitney U-test and Student’s T-test by comparing treated with non-treated cells. Flow cytometry of *T. cruzi* epimastigotes (**C**) and *T. brucei* PCF (**E**) non-treated (NT, black line) or treated for 16 hours with I-17 at 10 µM (red line) stained with propidium iodide before flow cytometry analysis. Cell population in G1 and G2 phases are indicated. Percentage of population from *T. cruzi* epimastigotes (**D**) and *T. brucei* PCF (**F**) in G1 and G2 cell cycle phases in presence (grey bars) or absence (empty bars) of I-17 are also displayed. The values are means ± standard deviation of triplicate experiments and were analyzed with Student´s t-test with *p < 0.05.

We observed aberrant cellular morphology of epimastigotes incubated with I-17. After 4 hours incubation with 3 µM of I-17, the parasites rounded up, as visualized by immunofluorescence for tubulin, which is mainly found in the parasite subpellicular array of microtubules (Fig. 2A). This morphology was observed in 40% of the population treated with 3 µM I-17 and close to 90% in parasites incubated with 10 µM I-17 (Fig. 2B). Morphology changes also occurred in *T. brucei* PCF incubated with I-17 (data not show). Flow cytometry analysis indicated that I-17 treatment increased the percentage of *T. cruzi* epimastigote (Fig. 2C and D) and *T. brucei* PCF (Fig. 2E and F) population in the G1 cell cycle phase, suggesting an arrest of replication in both species.

### I-17 induced eIF2α phosphorylation and protein synthesis arrest

**Figure 3:**
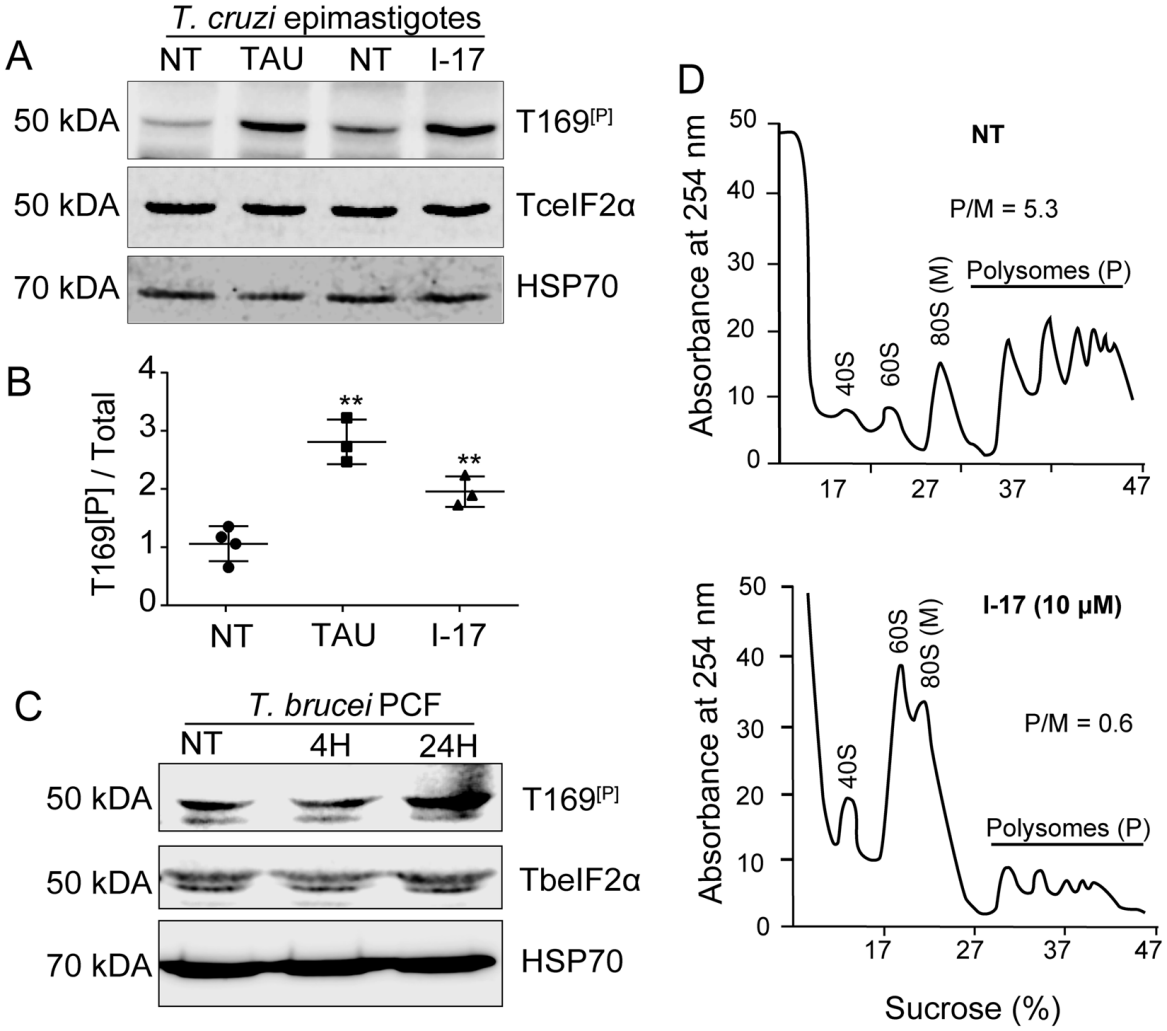
I-17 causes eIF2α phosphorylation and translational initiation impairment. (**A**) Total extracts of exponentially growing *T. cruzi* epimastigotes (NT), submitted to nutritional stress (TAU), or treated with 10 µM of I-17 for 4 hours were analyzed by immunoblotting using antibodies against the phosphorylated threonine 169 of eIF2α (T169^[P]^), anti-TceIF2α, and anti-HSP70. The original figures are depicted in as Supplementary Figure 2. (**B**) Ratio between phosphorylated (T169^[P]^) and the total TceIF2α intensity signal. The values are mean ± standard deviation of triplicate experiments and **indicates p < 0.01 calculated using the Student’s t-test. (**C**) Total extracts of exponentially growing *T. brucei* procyclics (NT) were submitted to 10 µM of I-17 for 4 or 24 hours and analyzed by immunoblotting using antibodies against the phosphorylated threonine 169 of eIF2α (T169^[P]^), anti-TceIF2α used to detect TbeIF2α, and anti-HSP70. (**D**) Polysome profiles in sucrose gradients measured at 254 nm of epimastigotes non-treated (NT) or treated with I-17 at 10 µM. The migrating position of the ribosomal subunits (40S and 60S), monosomes (80S) and polysomes (P) is indicated in each panel. The P/M ratios were obtained by measuring the graphic area under both fractions.

Phosphorylation of eIF2α at serine 51 decreases protein synthesis in eukaryotic cells submitted to different types of stress^20^. This phosphorylation allows cells to translate preferentially proteins that act in stress remedial response^30^. We have previously found that *T. cruzi* epimastigotes phosphorylate eIF2α when submitted to nutritional stress^31^. The parasite eIF2α is phosphorylated in the threonine 169 (T169^[P]^), which corresponds to the serine 51 of most eukaryotes. Therefore, we tested whether eIF2α was phosphorylated when parasites were exposed to I-17. As shown in Fig. 3A, I-17 treatment at 10 µM for 4 hours caused an increased phosphorylation of eIF2α detected by an specific antibody to T169^[P]^, in comparison to non-treated cells (NT), in a similar manner to nutritional stress caused by incubating parasites in a saline medium (TAU), mimicking the urine of the insect vector^32^. The increase of eIF2α phosphorylation was observed and quantified in three independent experiments, showing similar results (Fig. 3B). An increase in T169^[p]^ signal was also detected in PCF treated with I-17 for 24 hours (Fig. 3C).

These results suggest that I-17 could reduce general translation triggered by eIF2α phosphorylation. In fact, polysome profiling confirmed that less polysomes were present in cells treated with 10 µM I-17 in comparison with non-treated parasites (Fig. 3D). There was a large increase in RNA associated with the monosome (80 S) fractions compared to polysome fractions. The polysome to monosome ration (P/M) changed from 5.3 to 0.6 in control vs. I-17 treated samples respectively, indicating translation arrest at the initiation level. These data implicate that I-17 may be causing translation initiation arrest as a consequence of eIF2α phosphorylation.

### The effect of I-17 on *T. brucei* multiplication is dependent on eIF2α phosphorylation and on a specific kinase

**Figure 4:**
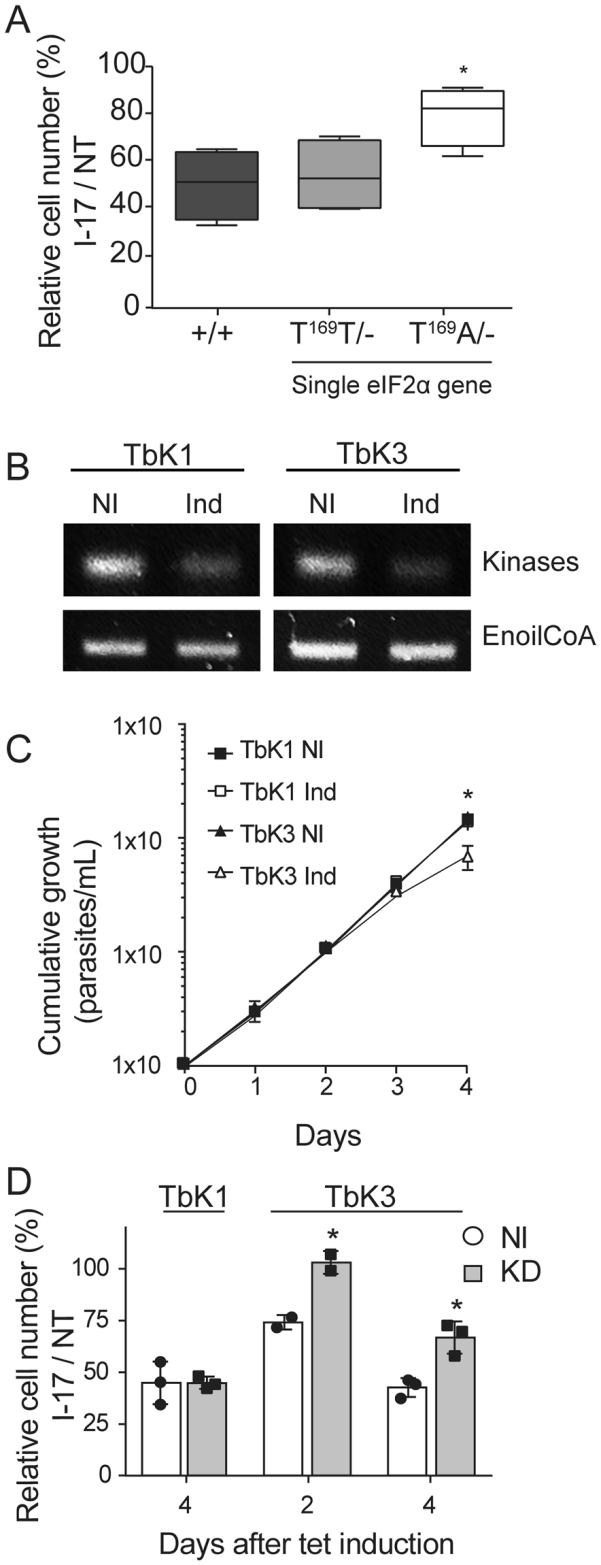
I-17 inhibition depends on phosphorylatable eIF2α and a specific eIF2α kinase. (**A**) Relative cell numbers after 24 hours were measured in percentages by dividing the total number of *T. brucei* BSF treated with 5 µM of I-17 by the cell number of the appointed lineages maintained without I-17 (NT). (+/+) corresponds to a parental BSF line, (T169T/−) to BSF containing a single wild type eIF2α gene, and (T169A/−) to BSF containing one alelle of eIF2α gene mutated for alanine at T169. The boxes are means ± min and max values of quadruplicate experiments. *Indicates p < 0.05 calculated using the Student’s t-test. (**B**) RT-PCR of total RNA extracted from PCF stably transfected using p2T7-177 with segments of TbK1 and TbK3 kinases after four days without (NI) or with tetracycline-induction for RNAi expression (Ind). The top panels show a PCR using primers for TbK1 and TbK3, and the bottom panels using primers for the enoyl-CoA as an expression control. The original gels are shown in the Supplementary Figure 3. (**C**) Cumulative growth curve of TbK1 (squares) and TbK3 (triangles) PCF lineages without (NI) or with tetracycline (Ind) for RNAi induction. The values in (C) are means ± standard deviation of triplicate and independent experiments. (**D**) Relative cell numbers in percentages measured as the ratio of total cell numbers of cultures treated with 3 µM I-17 and non-treated cultures (NT), using both TbK1 and TbK3 lineages non-induced with tetracycline (NI, empty bars) or after RNAi induction (Ind, grey bars). I-17 treatment was maintained for 48 hours and initiated 2 or 4 days after tetracycline addition. The values in (D) are means ± standard deviation of triplicate and independent experiments. *Indicates p < 0.05 calculated using the Student’s t-test.

To define a cause and effect relationship between eIF2α phosphorylation induction and I-17 inhibition of parasite multiplication, we examined the susceptibility of *T. brucei* BSF, in which one eIF2α allele was deleted and the other had threonine 169 replaced by alanine (T169A/−) to prevent phosphorylation. When comparing I-17 treated with non-treated cells (NT), expressed as relative cell numbers, the parasite strain expressing a non-phosphorylatable eIF2α (T169A/−) was consistently less susceptible to I-17 at 5 µM compared to the parasite line in which the endogenous eIF2α gene was replaced with wild type eIF2α (T169T/−), or with the parental strain (+/+) containing original alleles (Fig. 4A). This indicates that eIF2α phosphorylation is one, albeit possible not an exclusive, mediator of the anti-trypanosome inhibition induced by I-17.

The phosphorylation of eIF2α is regulated by four kinases in mammals^33^. Trypanosomatids genome contains three eIF2α kinases; K1, K2 and K3^34^. To identify which of these kinases could be involved in eIF2α phosphorylation by I-17, we generated knockdown lineages of PCF by RNAi for these three *T. brucei* kinases. We were able to obtain PCF lineages showing reduction of TbK1 and TbK3 RNA levels as seen by RT-PCR after induction of RNAi with tetracycline for 4 days (Fig. 4B). We could not obtain a conclusive knockdown of TbK2 (data not shown). As control, we found no changes in mRNA levels of enoyl CoA, a non-related enzyme. TbK1 and TbK3 knockdown lineages showed similar multiplication until 3 days after knockdown induction, but from the third to the fourth day of induction, TbK3 knockdowns replicated slightly less (Fig. 4C). Therefore, TbK1 and TbK3 lineages were tetracycline-induced for 2 or for 4 days previously to the treatment with 3 µM of I-17 for two more days. The different effect of I-17 was noticed by expressing the relative number of treated divided by non-treated cells (relative cell number), for TbK1 and TbK3 RNAi lineages induced (Ind) or not-induced (NI) (Fig. 4D). We observed that the relative cell number was 30% higher 2 or 4 days after induction of the TbK3 knockdown compared to the same lineage without RNAi induction. In contrast, TbK1 knockdown induction did not interfere with the inhibition of parasite multiplication caused by I-17. With these data, we demonstrated that, at least partially, TbK3 has a role on inducing inhibition of multiplication in *T. brucei* treated with I-17.

## Discussion

In this work, we tested 1,3-di-substituted urea analogs designed and synthesized to activate mammalian heme regulated inhibitor and found that among 25 different compounds, I-17, 3p and 6a inhibited *T. cruzi* epimastigotes proliferation (EC_50_ below 5 µM) with toxicity lower than mammalian cells. Compound I-17 also inhibited the intracellular replication of *T. cruzi* and the proliferative stages of *T. brucei*, BSF and PCF, with similar EC_50_ values. The selectivity to inhibit intracellular multiplication of *T. cruzi* was around 17-fold higher than host cell toxicity and occurred simultaneously with reversible parasitic cell cycle arrest and inhibition of translation, at least partially, in an eIF2α phosphorylation-dependent manner.

As these compounds were shown to present inhibitory activity against melanoma cells^26^, they could be targeting relevant molecules that would allow growth and resistance of transformed cancerous cells as well as parasites. Interestingly, cancer cells have to deal with oxidative stresses^35^, a capacity also critical for *T. cruzi* virulence^36^. It is noticeable that changes on the 4-aryloxycyclohexyl moiety bearing an *N*-(3-trifluoromethoxy) phenyl ring, designed in 3p, 6a, 3p, 6b di-substituted ureas showed better results against epimastigotes but at the cost of a lower selectivity relative to mammalian cells. I-17 was effective at concentration slightly smaller for intracellular amastigotes (1.2 ± 0.5 µM) compared to epimastigotes (3.4 ± 1.3 µM) and the host cell toxicity was variable among different cell lines and employed methodology, resulting in a selectivity index ranging from 17 to 9.5. Despite this relatively moderate selectivity, the action of I-17 could serve to probe the role of eIF2α in controlling *T. cruzi* infection.

Observations that I-17 promotes formation of enlarged epimastigotes with increasing numbers of cells in G1 phase could indicate that I-17 triggered a blockage in the DNA replication (S phase of cell cycle), arresting *T. cruzi* proliferation. Inhibition of replication and a shift in the percentage of G1:G2 cell cycle phases ratio also occurred in a similar way for PCF of *T. brucei*. Nevertheless, this is a well-known phenotype generated by several gene knockdowns obtained by RNAi in *T. brucei*, some of them affecting cell cycle^37^. Therefore, it is possible that changes in cell shape and cell cycle might have arisen as consequence of a general trypanosome stress response^38–40^.

We found that I–17 induced phosphorylation of *T. cruzi* and *T. brucei* eIF2α at threonine 169 residue (T169), which is functionally homologous to the Ser51 of eIF2α in mammalian and yeast cells. On those organisms, it is relatively well described how Ser51 eIF2α phosphorylation is responsible for delaying binding of methionine initiator tRNA to ribosomes and regulating protein synthesis during stressful conditions^41^. The T169 phosphorylation signal increased in both *T. cruzi* and *T. brucei* with time and was associated with decreased levels of polysomes, correlating growth inhibition with protein translation decrease. Consistently, *T. brucei* lineages containing eIF2α with T169 mutated to non-phosphorylatable alanine (T169A/-)^42^ were relatively more resistant to I-17 than control cells containing threonine, a phosphorylatable residue. There is a formal possibility that phosphorylation of eIF2α was consequence of stress induced by the compound rather than activation of parasite eIF2α kinase, as described for mammalian cells^21^. For example, a modified urea derivative was shown to interact with mammalian CXCR2 receptor preventing signaling^43^, and fluorinated 1,3-diarylureas were found to activate AMP-activated protein kinase, inhibiting cell-cycle progression and proliferation in colorectal and stem cells cancers^44^. This, however, is unlikely to occur in *T. cruzi* as eight closely related analogs (3b, 3n, 3d, 3j, 3k, 3l, 3t and NCPdCPU) which failed to activate mammalian eIF2α kinases also have no activity against parasites^23,26^.

We noticed that *T. brucei* BSF T169A/− was only partially resistant to I-17. This partial increase in resistance, i.e. cell numbers in presence of I-17 was closer but not equal to the number of cells from untreated cultures, implicates that I-17 may be interfering with other molecular targets mechanisms that affect *T. brucei* and possibly *T. cruzi* proliferation and/or survival. This partial protection in the non-phosphorylatable lineage (T169A/−) could also be explained if parasite cells required some level of eIF2α phosphorylation for stress protection triggered by I-17. Alternatively, T169A/− eIF2α lineage could have a different set of expressed proteins, as consequence of different translational regulation. Nevertheless, parasite survival could additionally be affected by preventing eIF2α phosphorylation, which is known to affect fate decisions in cancerous cells^21^.

Three eIF2α kinases homologues have been described in Trypanosomatids^34,45–47^. Here, we further demonstrated that RNAi knockdown of *T. brucei* eIF2α kinase 3 (TbK3)^33^, increased resistance of PCF to I-17. This suggests a role for TbK3 kinase as responsible for eIF2α phosphorylation triggered due to I-17 treatment, at least in *T. brucei*. TbK3 has a conserved eIF2α kinase domain^34^ and it was shown to be essential in regulating spliced leader expression by affecting the transcription factor TRF4 after endoplasmic reticulum stress^47^. Whether TbK3 can directly phosphorylate eIF2α or targets TRF4 remains to be demonstrated for both trypanosomes, as the linkage between these pathways is still obscure. Furthermore, we could speculate that the presence of I-17 could activate TbK3, directly or indirectly, inducing eIF2α phosphorylation and causing translation arrest, further preventing spliced leader transcription. This would cause a general growth arrest since spliced leader is added to most of the pre-mRNA messengers to allow nuclear export and translation^48^. This type of response was described as an unfolded protein response-like that upon persistence could lead to annexin binding to the parasite and DNA fragmentation, independently of caspase activations^49^, further leading to cell death. Depletion of TbK1, a homologous of GCN2 kinase involved in recognizing amino acid starvation on different organisms, including *Leishmania* parasites^46^, did not change the resistance to I-17. We were not able to obtain knockdowns of TbK2, the other eIF2α kinase of *T. brucei*, so its role in I-17 could not be evaluated.

It is relevant that some 1,3-diarylureas have been tested with some success against flatworm parasites such as *Schistosoma japonicum*^50^ and *Schistosoma mansoni*^51^, although no targets were identified in those cases. Also, a family of 1,3-diarylureas with a 4-aminoquinaldinyl as one of the aryl rings was shown to be active against chloroquine sensitive and resistant strains of *Plasmodium falciparum*^52^, the causative agent of malaria. 1,3-diarylureas have two available cyclic rings that can be readily substituted by various functional groups, allowing the synthesis of several improved compounds^53^. The structure of I-17 could be used to design *Trypanosoma* kinases activators capable of interfering with eIF2α stress responses, potentially useful in combinatory therapies with current drugs, such as benznidazole and nifurtimox, known to be potent oxidative stress inducers^8,54^.

## Conclusion

An 1,3-di-substituted urea (I-17) is cytostatic to trypanosomatids partially by promoting phosphorylation of eIF2α which causes protein synthesis arrest. Parasites lacking a phosphorylatable eIF2α site or with reduced levels of eIF2α-kinase 3 were more resistant to treatment. The compound I-17 also generates an aberrant cell morphology and cell cycle arrest. These findings indicate that further exploring the stress response through eIF2α regulation could help provide basis to obtain more selective compounds against *Trypanosoma* parasites compared to mammalian cells, especially through quantitative structure activity relationship studies, for example by elucidating the atomic structure of parasite eIF2α kinases.

## Material and Methods

### Ethics Statement

The procedures used in this work were approved by the “Comite de Ética em Pesquisa da Universidade Federal de São Paulo” under protocol 2666291015. All methods were performed in accordance with relevant guidelines and regulations approved by the Universidade Federal de São Paulo.

### Compounds synthesis and preparation

Discovery, biological and analytical characterization of 1,3-diarylurea compounds 1-(benzo [d][1,2,3] thiadiazol-6-yl)-3-(3,4-dichlorophenyl) urea (BTdCPU) and 1-(2-chloro-5- nitrophenyl)-3-(3,4-dichlorophenyl) urea (NCPdCPU) was reported by^23^. BTdCPU is an activator of the activated heme regulated kinase leading to eIF2α phosphorylation, while NCPdCPU is inactive against this target. Synthesis and chemical characterization of cHAUs compounds and *in vitro* and *in vivo* evaluation have been reported elsewhere^24,26^. Structures of these compounds are shown in Supplementary Figure 1.

### Parasites and cell cultures

The bloodstream form (BSF) of *T. brucei* Lister 427 strain was cultivated in HMI-9 medium in the presence of 10% fetal bovine serum (FBS) and Serum Plus (SAFC Biosciences) containing 59 mg/mL penicillin and 133 mg/mL streptomycin at 37 °C in a CO_2_ incubator^55^. We used the *T. brucei* EATRO 1125 parental lineage (+/+), or derivatives in which one of the alleles of endogenous gene coding for eIF2α was replaced by blasticidin resistance coding sequence and the other allele by either the wild type eIF2α gene (T169T/−), or a mutated version in which threonine 169 was replaced by alanine (T169A/−) in a cassette containing the aminoglycoside 3′-phosphotransferase coding sequence that allow resistance to Geneticin G418^56^. These strains were maintained as described above by replacing the FBS by rabbit serum (Sigma-Aldrich) and the modified lineages were growth in the presence of 2.5 µg/mL Geneticin-G418 and 5 µg/mL blasticidin (both from Thermo Fisher Fisher). The procyclic form (PCF) of *T. brucei* Lister 427 and its derivative 29–13^57^ were cultivated at 28 °C in SDM-79 medium^58^ supplemented with 10% FBS and 7.5 µg/mL hemin. For 29–13 PCF, 50 µg/mL hygromycin B and 15 µg/mL Geneticin-G418 were also added. BSF and PCF were diluted to 2 × 10^5^/mL and incubated in medium containing the indicated concentrations of compounds previously diluted in DMSO. Epimastigotes of *T. cruzi* (Y strain) were cultivated at 28 °C in autoclaved liver infusion tryptose (LIT) medium supplemented with 1 µg/mL hemin, 10% FBS, 4 mg/mL glucose, 59 mg/mL penicillin and 133 mg/mL streptomycin^59^. The epimastigotes were diluted to 2 × 10^6^/mL and incubated with the indicated concentrations of compounds. Multiplication of *T. brucei* and *T. cruzi* were assessed by counting the number of parasites after 24, 48 and 72 hours respectively, using hemocytometer in three independent experiments.

Tissue culture derived *T. cruzi* trypomastigotes (TCT) were obtained from supernatants of infected LLC-MK_2_ (Rhesus monkey kidney epithelial cells, ATCC CCL-7) cultivated in low glucose Dulbecco’s Modified Eagle Medium (DMEM, Thermo Fisher Scientific) supplemented with 3.7 g/L sodium bicarbonate, 10% FBS, 59 mg/mL penicillin and 133 mg/mL streptomycin at 37 °C in a CO_2_ incubator. The parasites were collected by centrifugation at 1500 g for 5 min, incubated for at least 60 min at 37 °C and the TCT enriched supernatants were collected to be used for new infections. LLC-MK_2_, L6 rat myoblasts (ATCC CRL-1458) and U-2 OS (Banco de Células do Rio de Janeiro, Brazil) maintained in the same medium were used for evaluating the progression of *T. cruzi* infection. The cells (1 × 10^5^) were seeded onto 13 mm glass coverslips in 24 well plates and incubated at 37 °C. The next day, 5 × 10^5^ TCT in 500 µL were added to each well and incubations proceeded for 2 hours. Wells were washed twice with PBS and incubation continued in the presence of culture medium with the indicated amounts of each compound. After 72 hours, the cells were fixed in Bouin solution (acetic acid 5%, formaldehyde 9% and picric acid 0.9%) for 5 min and washed four times with PBS before staining for 1 hour with Giemsa solution (previously diluted 1:10 in tap water and filtered). Glass coverslips were dehydrated in acetone, acetone with xylol, and only xylol before mounted in glass slides covered with Entellan mounting medium (Sigma-Aldrich). Coverslips were visualized under an optical microscope and the number of intracellular amastigotes assessed in one hundred infected cells.

### Parasite viability assays and EC_50_ determination

*T. cruzi* epimastigotes at 1 × 10^7^/mL in 50 µL were incubated in 96-wells plates with the same volume of compounds pre-diluted in 50 µL of LIT medium for 48 hours at 28 °C. LLC-MK_2_ cells (3 × 10^3^) in 100 µL of medium were added to 96 well plates. After 5 hours, the medium was replaced by fresh medium containing serial dilutions of test compounds and incubations proceeded for 72 hours at 37 °C. In both cases, 10 µL of AlamarBlue (Thermo Fisher Scientific) was added and the plates were further incubated for 18 hours at 28 °C for epimastigotes and 4 hours at 37 °C for mammalian cells. Absorbance at 570 nm and 600 nm were measured by using a SpectraMax M3 (Molecular Devices). The differences between absorbance at 570 nm and 600 nm for each well was normalized against a blank value for medium without parasites and lysed cells. Alternatively, L6 rat myoblast cell death and lysis were determined using the cytotoxicity Detection Kit Plus LDH Version 6 (Roche) after 72 hours incubation with the indicated compounds using the manufacturer protocol. Data was analyzed using GraphPad Prism 6.01 software with logarithmic nonlinear regression to generate a response curve fitting and determine the effective concentration that affects 50% of cells (EC_50_) or 90% of cells (EC_90_). Each compound was tested in triplicates in at least two independent experiments, with exceptions appointed on Table 1. Selective index was estimated by the ratio between LLC-MK_2_ EC_50_ and epimastigotes EC_50_ on Table 1.

### High content analysis of *T. cruzi* infection

To evaluate resistance to I-17 during intracellular amastigote replication, we used a system of high content imaging analysis that allowed processing of thousands of cells to robustly evaluate inhibitory effects on intracellular *T. cruzi* multiplication simultaneously to evaluation of host cell toxicity. Stock solution of I-17 was diluted to 5.7 mM in DMSO. Before use, the compound was transferred onto 384-well polypropylene stock plates (Greiner Bio-One) and further diluted into 10-point dose response curve (2-fold serial dilution) in neat DMSO. These stock plates were kept frozen at −20 °C and protected from light.

U-2 OS cells were plated at 800 cells per well in 40 μL of medium and incubated for 24 hours at 37 °C. The cells were then infected with 10 μL of trypomastigotes suspension at 1.5 × 10^6^/mL, harvested from the supernatant of LLC-MK_2_ cultures. The next day, serially diluted I-17 solutions were transferred into intermediate plate containing culture medium in order to decrease DMSO and I-17 highest concentration to 6% (v/v) and 342 µM, respectively. Finally, ten microliters of compound solution were transferred onto assay plates, yielding a final concentration of 1% DMSO (v/v) in a final volume of 60 μL, starting from 57 µM of I-17. DMSO-treated wells containing non-infected and infected cells were used as positive and negative controls, respectively. After 4 days at 37 °C, the cells were fixed with 4% paraformaldehyde diluted in PBS (PFA), stained with Draq5 (Biostatus) for 30 minutes at room temperature in the dark, and six images per well, totalizing more than a thousand cells, were acquired using the high content imaging system INCell 2200 (General Electric) at 20× agnification. Images were analyzed by using Investigator, version 1.6 (General Electric) for identification, segmentation and quantification of host cell nucleus, cytoplasm and intracellular parasites. The analysis provided output data for all images as the total number of cells, total number of infected cells and the total number of intracellular parasites. The infection ratio was obtained by considering the number of infected cells relative to the total number of cells and was normalized to the infections obtained in the presence of 1% DMSO (negative control) and non-infected cells to calculate the normalized antiparasitic activity, according to the following formula: Normalized Activity (NA) = [1− (Average IR_N_ − Average IR_T_)/(Average IR_N_ – Average IR_P_)] × 100. Where IR_N_ is the infection ratio of negative control wells, IR_P_ is the infection ratio of positive control wells and IR_T_ is the infection ratio of test compound wells. The final results of compound activity define the percentage of *T. cruzi* free cells normalized to control values.

Dose-response curves were processed with Graphpad Prism software, version 6, for generation of sigmoidal dose-response (variable slope) nonlinear curve fitting and determination of EC_50_ values by interpolation. Percentage of host cell survival was calculated as a relation between the number of cells on treated wells relative to the number of cells in non-treated wells as an indicative of cytotoxicity and replication inhibition.

### Immunofluorescence

Exponentially growing *T. cruzi* epimastigotes were diluted in medium to 8 × 10^6^/mL and incubated with the indicated compounds. At the end of treatments, cells were collected by centrifugation at 2000 g for 2 min, washed and resuspended in PBS before addition to glass slides (Tekdon Inc.) previously coated with 0.01% poly-L-lysine (Sigma-Aldrich) for 20 min. After 5 min, the unattached parasites were removed, and the slides were incubated with PFA, washed with PBS and permeabilized with 0.1% Triton X-100 in PBS for 5 min. The slides were then incubated 20 min with 1% BSA in PBS, followed by 1 hour incubation with a mouse antibody against β-tubulin C-terminal peptide^45^ diluted 1:1000 in blocking solution. The slides were washed with PBS, incubated 1 hour in the dark with anti-rabbit IgG antibody coupled to Alexa Fluor 488 (Thermo Fisher Scientific) at 1:1000 in PBS and 10 µg/mL 4′,6-diamidino-2-phenylindole (DAPI). Slides were washed twice with PBS and mounted with Prolong Gold Antifade Reagent (Thermo Fisher Scientific). Images were acquired using a Hamamatsu Orca R2 CCD camera coupled to an Olympus BX-61 microscope equipped with an x100 plan Apo-oil objective (NA 1.4). AutoQuant X2.2 software (Media Cybernetics) was used to perform blind deconvolution.

### Cell cycle analysis

After the indicated treatments, parasites were collected by centrifugation at 2000 g for 2 min, washed in PBS, fixed with 50% cold methanol for 10 min and washed in PBS again. To eliminate RNA background staining, the cells were incubated with RNAse (10 µg/mL) for 30 min at 37 °C, washed once more with PBS and diluted in 1 mL of PBS containing 10 µg of propidium iodide for 5 min. Parasites were quickly washed twice with PBS and analyzed in a BD Accuri C6 (BD Sciences) flow cytometry using FL-2 channel for fluorescence detection.

### Immunoblotting

Parasites treated as indicated were collected by centrifugation at 2000 g for 5 min and washed once in TBS (50 mM Tris-HCl pH 7.5, 150 mM NaCl) before addition of Laemmli buffer and heat denaturation at 95 °C for 5 min. Electrophoresis in polyacrylamide gels containing SDS and transfer to nitrocellulose membranes were performed by standard procedures. The membranes were stained with 0.5% Ponceau S in 3% acetic acid and cut separating the portion containing proteins with mass above and below 60 and 40 kDa. All membranes pieces were blocked with TBS containing 0.1% Tween 20 (TBS-T) and 5% BSA overnight. Membrane pieces from 40 kDa to 60 kDa were incubated for 3 hours with affinity purified antibodies reacting with the phosphorylated T169 (T169^[P]^) of *T. cruzi* eIF2α^31^ diluted 1:125 in blocking buffer. The primary antibody was washed 3 times in TBS-T for 10 min and incubated for 1 hour with anti-rabbit IgG peroxidase-conjugated antibody (Thermo Fisher Scientific) diluted 1:20000 in TBS. Membranes were washed three times in TBS-T for 10 min and bound antibodies detected by ECL (EMD Millipore) by using an Odyssey Fc System (LI-COR Biosciences). The same membrane pieces were re-probed with an antiserum (anti-TceIF2α) obtained by immunization of mice with a recombinant *T. cruzi* eIF2α expressed in *Escherichia coli*^31^. The serum was diluted 1:2000 in PBS containing 5% non-fat dry milk and after 1 hour incubation the membranes were washed three times with TBS-T followed by 1 hour incubation with anti-mouse IgG IRDye 800 (LI-COR Biosciences) 1:10000 in PBS. Membranes were washed three times with TBS-T and bound antibodies detected as described above. The nitrocellulose pieces from 60 kDa to the membrane upper edge were incubated with rabbit serum anti-HSP70^60^ diluted 1:10000 in blocking buffer for 1 h, washed three times with TBS-T and incubated with secondary anti-rabbit IgG IRDye 680 (LI-COR Biosciences) 1:10000 on TBS for 1 hour before another three washes in TBS-T and detection with Odyssey Fc System.

### Polysome profiling

At least 1 × 10^9^ of exponentially growing parasites were treated with the indicated concentrations of inhibitors. After 24 h, cycloheximide was added to 100 µg/mL and the cells maintained at 28 °C for 10 min before further incubation on ice for 10 min, centrifuged 5 min at 2000 g at 4 °C, washed in ice-cold PBS containing the same concentration of cycloheximide and diluted in 300 µL of ice-cold Buffer A (10 mM Tris-HCl, pH. 7.4, 300 mM KCl, 10 mM MgCl_2_, 1 mM dithiothreitol and 100 µg/mL cycloheximide). A small drop of Triton X-100 was added on the lateral side of tube to reach 1% (v/v) and the tubes were repeatedly mixed by inversion for about 5 min. The lysates were centrifuged for 3 min at 5000 g at 4 °C and the supernatant collected and added to a new tube. Stocks of heparin at 100 mg/mL and NaCl at 5 M were added to 1 mg/mL and 150 mM respectively. The corresponding volume of the samples containing the equivalent of ten absorbance units at 260 nm were then loaded on the top of a 7% to 47% sucrose gradient in Buffer A prepared using a Gradient Master (Biocomp). The tubes were centrifuged at 200,000 g for 2.5 hours in a Beckman SW41 rotor at 4 °C. The gradient was collected from the top by injecting in the bottom a continuous flow of 60% sucrose at 1 mL/min using an Econo Gradient Pump (Bio-Rad) and the fractions monitored by reading the absorbance at 254 nm.

### *T. brucei* eIF2α-kinases knockdown

A small fragment of the coding sequence between residues 2595 and 3066 of the *T. brucei* eIF2α-kinase 1 (TbK1, tritrypdb.org: Tb927.11.7210) and residues 1479 to 1989 eIF2α-kinase 3 (TbK3, tritrypdb.org: Tb927.6.2980) were PCR amplified from genomic DNA of *T. brucei* EATRO 1125 strain using the following oligonucleotides: TbK1XbaFow (5′-GCTCTAGATGTGAGTCCATTGAACCGGG) and TbK1XhoRev (5′-GCTCGAGTAAACGTCCTGAGGCCGAAC) and TbK3XbaFow (5′-GCTCGAGAACTCCAGCACAATCCTCCG) and TbK3XhoRev (5′-GCTCGAGAACTCCAGCGCAATCCTCCG). The fragments were digested with *Xho*I and *Xba*I and cloned into *Xho*I and *Xba*I restriction sites of the p2T7–177 plasmid^61^ to generate inducible knockdowns in PCF 29–13 strain^57^. The obtained plasmids were linearized with *Not*I, purified with QIAquick Gel Extraction Kit (Qiagen), diluted in 200 µL of Zimmerman’s Post Fusion Medium (ZPMF) containing 10^8^ PCF of 29–13 strain, and pulsed in 0.2 mm cuvettes with X-014 program on Amaxa Nucleofactor (Lonza). The cells were then diluted in SDM-79 medium and selection was done by adding 2.5 µg/mL phleomycin. After selection, expression of double-strand RNA for TbK1 or TbK3 knockdown induction was obtained by adding 1 µg/mL tetracycline daily.

### RT-PCR

Total RNA was extracted from the parasites using Trizol (Thermo Fisher Scientific) and treated with 2 U of DNAse I per µL for 30 min at 37 °C. The enzyme was inactivated by 10 min incubation at 75 °C and 10 µg of the denatured RNA was incubated with 1 µM of oligo-dT, cooled on ice, and then incubated for 50 min at 50 °C with Superscript III reverse transcriptase (Thermo Fisher Scientific) in the presence of the recommend buffer, 5 mM dNTP, 2 mM DTT and 40 U RNase out (Thermo Fisher Scientific) in a final volume of 50 µL. The enzyme was inactivated at 70 °C for 15 min and the produced cDNA treated with RNAse H (2U) at 37 °C for 20 min. A PCR reaction was performed using 25 cycles at 62 °C for annealing using the following pair of oligonucleotides: TbK1Fq (5′-TCCGGAGAGTGTCAGCGGGT)/TbK1Rq (5′-TGTGGCTGAGAGCGGCCAAAC), and TbK3Fq (5′-GGGGTGAGTGGGAAAGGACGTG)/TbK3Rq 5′-GCCGAGGCACCGAAAGTCCC) to amplify the residues 788 to 985 of TbK1Fq and residues 424 to 619 of TbK3, respectively. Amplification of enoyl-CoA, a non-related protein of *T. brucei*^62^, was performed as a control using Enoyl_CR3_F (5′CGCATATGTTACGCTCCTGTGCACTTTTA) and Enoyl-CR3_R (5′CGCTCGAGCTACGAATTGGTGAAGAACGGTTT).

## Electronic supplementary material

Supplementary information
