# Supplementary material for: Identification of di-substituted ureas that prevent growth of trypanosomes through inhibition of translation initiation
Source: Sci Rep. 2018 Mar 20;8:4857. doi: 10.1038/s41598-018-23259-9 (PMC5861040; doi:10.1038/s41598-018-23259-9)
Supplement: Supplementary file 1 — Supplementary information [file 41598_2018_23259_MOESM1_ESM.doc]

SUPPLEMENTARY INFORMATION

**Identification of di-substituted ureas that prevent growth of trypanosomes through inhibition of translation initiation**

Fabricio Castro Machado^1^, Caio Haddad Franco^1,3^, Jose Vitorino dos Santos Neto ^2^, Karina Luiza Dias-Teixeira^2^, Carolina Borsoi Moraes^3,4^, Ulisses Gazos Lopes^2^, Bertal Huseyin Aktas^5^, Sergio Schenkman^1^

^1^Departamento de Microbiologia, Imunologia e Parasitologia, Escola Paulista de Medicina, Universidade Federal de São Paulo, 04039-032, São Paulo, SP, Brazil

^2^Laboratório de Parasitologia Molecular, Instituto de Biofísica Carlos Chagas Filho, Universidade Federal do Rio de Janeiro, Rio de Janeiro, RJ, Brazil

^3^Instituto Butantan, São Paulo, SP, Brazil

^4^Departamento de Microbiologia, Instituto de Ciências Biomédicas, Universidade de São Paulo, São Paulo, SP, Brazil

^5^ Hematology Laboratory for Translational Research, Department of Medicine, Brigham and Women’s Hospital and Harvard Medical School, 75 Francis Street, Boston, MA, 02115, United States

Correspondence and request to materials should be addressed to B.H.A. (huseyin_aktas@hms.harvard.edu) or S.S. (sschenkman@unifesp.br)

SUPPLEMENTARY MATERIAL

**Supplementary Figure 2**

Original gels corresponding to the Figure 3A and 3C. An extra lane corresponding to an additional control (NT) was present in the original gel for 3A and two unrelated sample was also presented in the original gel for 3C. Due to limitations of the available antibodies against the phosphorylated T169 and to cross reactions against other possible phosphorylated proteins, we cutted the nitrocellulose membranes in the 60 kDa and above the 40 kDa markers.

Figure 3A

Figure 3C


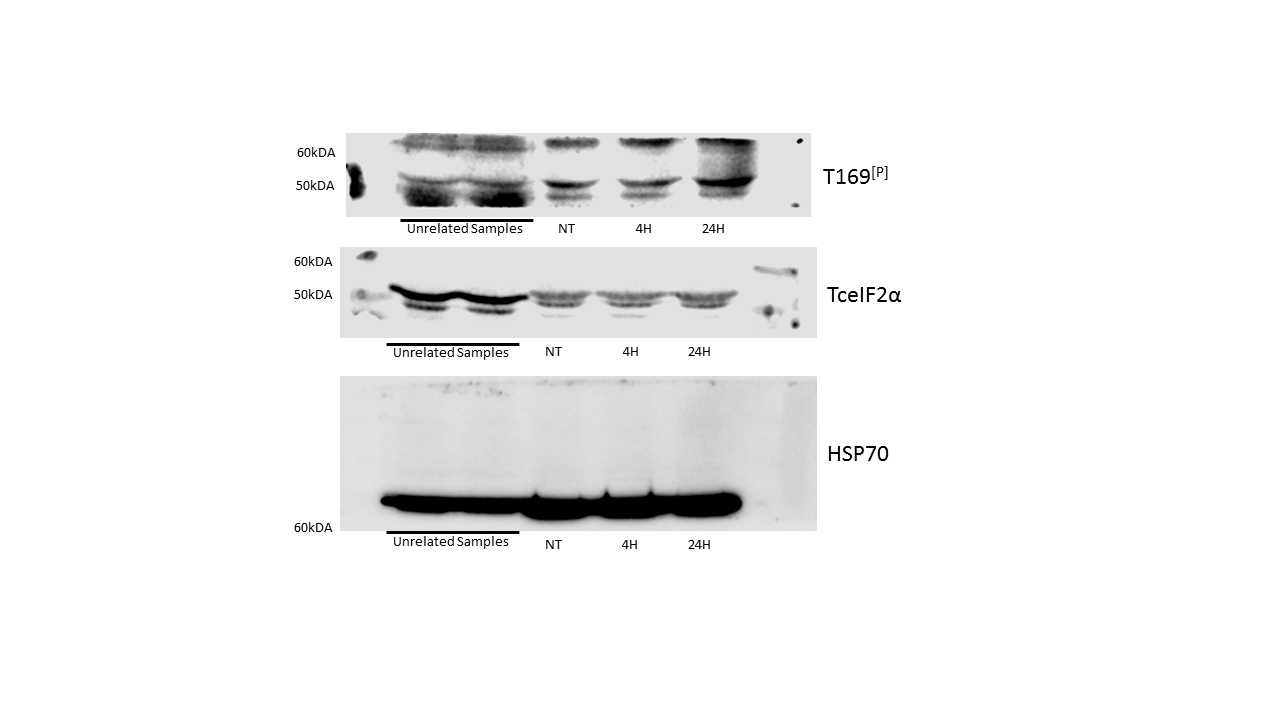


**Original figures used to build figure 4B.** (A). Agarose gel electrophoresis stained with ethidium bromide containing samples of RT-PCR to demonstrate the decrease in the expression of TbK1 and TbK2. The original experiment also employed the cell lines for TbK2, and we were not able to confirm the reduction in expression. The gel also shows control reactions made in the absence of reverse transcriptase (RT-) in opposition to the normal situation (RT+). The red boxes indicate the regions used in the final figure. The numbers at left indicate the size of markers in the lamda DNA digested with HindIII.

(B). Original agarose gel electrophoresis used as the positive control (Enoyl-CoA).
